# A new and interesting earthworm species from Madagascar (Crassiclitellata, Kynotidae) with an updated key to the *Kynotus* Michelsen, 1891 species

**DOI:** 10.3897/zookeys.1286.196760

**Published:** 2026-07-29

**Authors:** Csaba Csuzdi, Malalatiana Razafindrakoto, Yong Hong

**Affiliations:** 1 Independent researcher, 39 Kenderesi str., Piliscsaba, Hungary Université d’Antananarivo, Laboratoire des Radio-Isotopes (LRI) Antananarivo Madagascar; 2 Université d’Antananarivo, Laboratoire des Radio-Isotopes (LRI), Antananarivo, Madagascar Department of Plant Medicine, College of Agriculture & Life Science, Jeonbuk National University Jeonju Republic of Korea; 3 Department of Plant Medicine, College of Agriculture & Life Science, Jeonbuk National University, Jeonju 54896, Republic of Korea Unaffiliated Piliscsaba Hungary

**Keywords:** Barcoding, Malagasy Region, Megadrili, Oligochaeta

## Abstract

A new effort in 2025 to collect earthworms in northern Madagascar resulted in the discovery of a new species of *Kynotus*, a genus belonging to the endemic family Kynotidae, as well as the rediscovery of *K. darwinii*, the type species of *Kynotus*. The latter species had not been collected since its original reports in the late 19^th^ century. With the description of the new species, *Kynotus insularis***sp. nov**., the family Kynotidae now has 23 species. New DNA barcode sequences were generated for both species, confirming their distinctness and further demonstrating the high efficiency of the COI barcode region in identifying *Kynotus* species. An identification key to the currently known kynotid species is also presented.

## Introduction

The island of Madagascar is one of the largest in the world, covering an area of approximately 587,000 km^2^. It is an ancient fragment of the Gondwanaland supercontinent and lies just 400 km off the African coast, and its unique fauna has always been the focus of research (Andriamialisoa and Langrand 2022). The exploration of the earthworm fauna also began relatively early, when the Swiss naturalist Conrad Keller (1887) drew attention to the diversity of Madagascar’s earthworm fauna in his book and described a new species, *Geophagus darwinii* Keller, 1887 (= *Kynotus darwinii*). Four years later, Michaelsen (1891) reported a new earthworm species from Madagascar, *Kynotus madagascariensis* Michaelsen, 1891, which was aclitellate but with developed inner genital organs. This species was so peculiar that Michaelsen placed it in his newly erected genus *Kynotus* Michaelsen, 1891. Although not directly stated, the genus name may have derived from the Greek words κύων (dog) and ὠτός (ear, in the genitive form), meaning “ear of a dog”. This may refer to the eversible claspers (bursa propulsoria) of kynotids, which, when everted, resemble ears. Later, Michaelsen (1897) recognized that his genus *Kynotus* was identical to *Geophagus* as established by Keller (1887). However, because *Geophagus* Keller, 1887 was preoccupied by *Geophagus* Heckel, 1840 (a genus of cichlid fishes), *Kynotus* remained the valid name. Nevertheless, an examination of the type specimens of *Geophagus darwinii* Keller, 1887 by Michaelsen (1897) revealed that this species is a senior synonym of *Kynotus madagascariensis* Michaelsen, 1891, the type species of the genus *Kynotus* (Michaelsen 1897). Thereafter, research on Malagasy earthworms lapsed and remained largely dormant until 2008, when a new initiative, the Faune-M project, was launched with support from the French Institute of Biodiversity to investigate the island’s earthworm fauna. During this project, collections were made at numerous localities across the island, resulting in the description of six new *Kynotus* species (Razafindrakoto et al. 2011; Csuzdi et al. 2012; Razafindrakoto et al. 2017). Since 2015, researchers from South Korea have also joined the project, leading to the description of three additional *Kynotus* species (Csuzdi et al. 2017).

However, the exploration of the Malagasy earthworm fauna is still far from complete. This is clearly demonstrated by the results obtained after fieldwork resumed following the COVID-19 pandemic. In addition to the new *Kynotus* species described here, we collected the type species of the genus, *K. darwinii*, for the first time during our collection efforts.

## Materials and methods

### Earthworm collection and examination

The earthworms were collected by digging and hand-sorting. The collected worms were killed in 50% ethanol and preserved in either 96% ethanol or 4% formaldehyde. The holotype and paratypes of the new species are deposited in the Zoological Collection of the Eszterházy Károly Catholic University (**EKCU**) and the National Institute of Biological Resources, Korea (**NIBR**). Parallel samples suitable for DNA studies are deposited in the collection of Jeonbuk National University, Korea (**JBNU**).

The collected specimens were examined under a stereomicroscope and internal organs were studied through dorsal dissection. The genital setae were removed and placed on a cavity slide. After clearing the surrounding tissue, they were mounted in Euparal for further examination under a light microscope. The backgrounds of the captured microphotographs were subsequently refined using Gemini 3 AI.

### Molecular methods

Total genomic DNA was extracted from the tail of the body segments of a single individual using the DNeasy Blood & Tissue Kit (Qiagen, USA) following the manufacturer’s protocol. Sequencing adapters were ligated to both ends of the fragmented DNA using the TruSeq DNA Nano Library Prep Kit (Illumina Inc., USA) to construct a paired-end sequencing library. Filtered reads were assembled using a two-pass reference-assisted strategy to recover the COI gene fragment. To improve annotation accuracy, gene boundaries were further refined through BLAST searches against the NCBI database.

In addition to the newly generated sequences, supplementary data were retrieved from the GenBank and the BOLD Systems v5 database (Ratnasingham and Hebert 2007). The corresponding GenBank accession numbers or BOLD ID numbers are indicated next to each sequence in the phylogenetic tree.

COI sequences were aligned using ClustalW (Thompson et al. 1994) with default parameters as implemented in MEGA 7 (Kumar et al. 2016). The final alignment was 654 bp in length and contained no internal gaps. The best-fitting nucleotide substitution model (TIM2+I+G) was selected using ModelFinder (Kalyaanamoorthy et al. 2017), implemented in the IQ-TREE web server, based on the Akaike information criterion (AIC; Akaike 1973) and the Bayesian information criterion (BIC; Schwarz 1978). Bayesian analysis was performed using Mr.Bayes v. 3.2.7 (Ronquist et al. 2012) with the closest substitution model GTR+I+G. Each of the two independent runs was set to 10 million generations sampling a tree every 1,000 generations. After removing the first 2,000 trees as burn-in, the remaining trees were combined and summarized on a 50% majority-rule consensus tree. The resulting tree was visualized using FigTree v. 1.4.2 (Rambaut 2014). Interspecific genetic distances were calculated in MEGA 7 using the K2P distance method.

The Assemble Species by Automatic Partitioning (ASAP) species delimitation method was used to test morphologically defined species boundaries (Puillandre et al. 2021). Analyses were conducted using the Spart Explorer web platform (https://spartexplorer.mnhn.fr/) with the default parameter settings.

## Taxonomy

### Family Kynotidae Michaelsen, 1891

**Genus *Kynotus* Michaelsen, 1891**

#### Kynotus darwinii

Taxon classification

Animalia

CrassiclitellataKynotidae

(Keller, 1887)

10566BA6-84A2-594C-A766-F7A06D881691

Figs 1, 2

Geophagus darwinii Keller, 1887: 248.
Kynotus madagascariensis Michaelsen, 1891: 207.
Kynotus darwini: Michaelsen 1897: 244; Michaelsen 1900: 456.

##### Material examined.

Madagascar – Diana District • EKCU-Olig-38, 2 aclitellate ex.; NIBRIV0000934123 1 aclitellate ex.; JBNU0010, 1 aclitellate ex. (GenBank accession no. PZ269093); Ambohimena, • Ambanja; 13°36'57"S, 48°24'31"E; leg. M. Razafindrakoto; 15 Feb. 2025.

**Figure 1.**
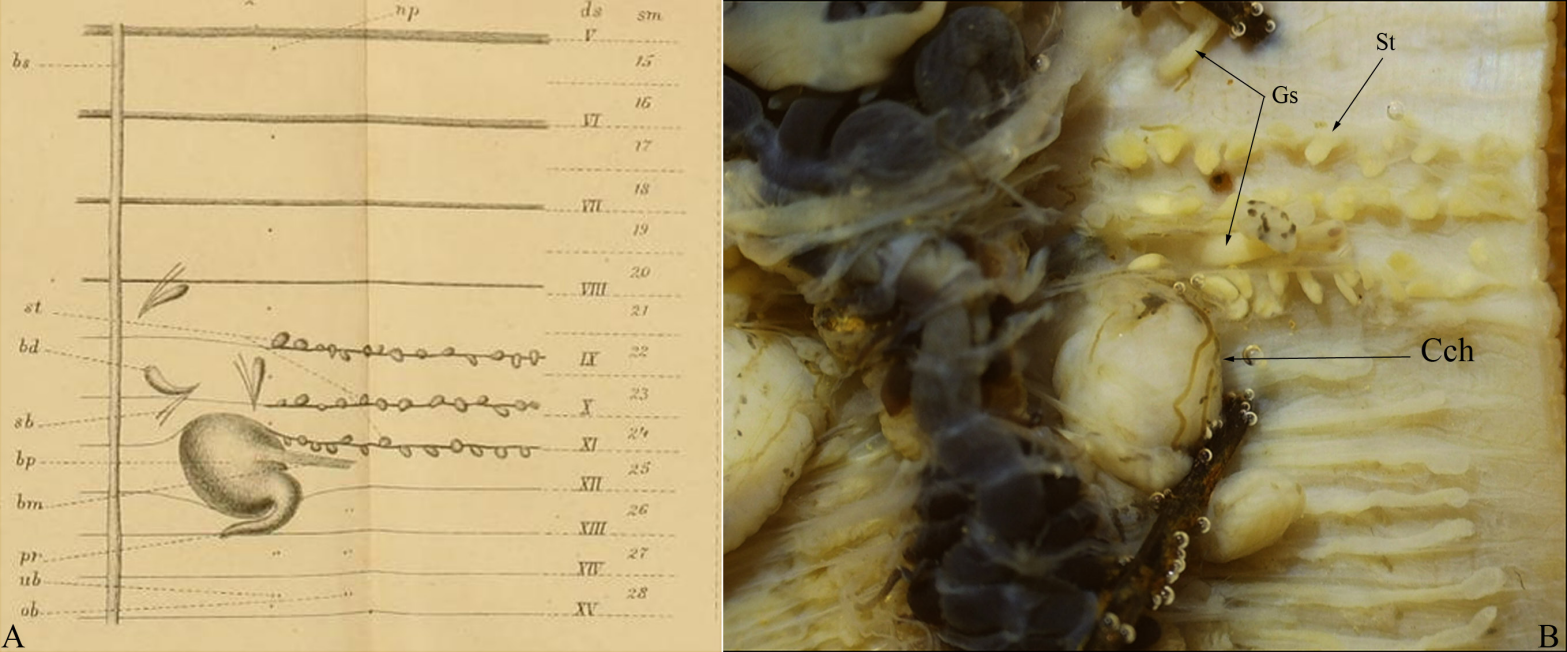
*Kynotus darwinii* (Keller, 1887). **A**. Spermatheae from Michaelsen (1891); **B**. Spermathecae of our specimen. Abbreviations: Gs = genital setae, St = spermathecae, Cch = copulatory chamber.

**Figure 2.**
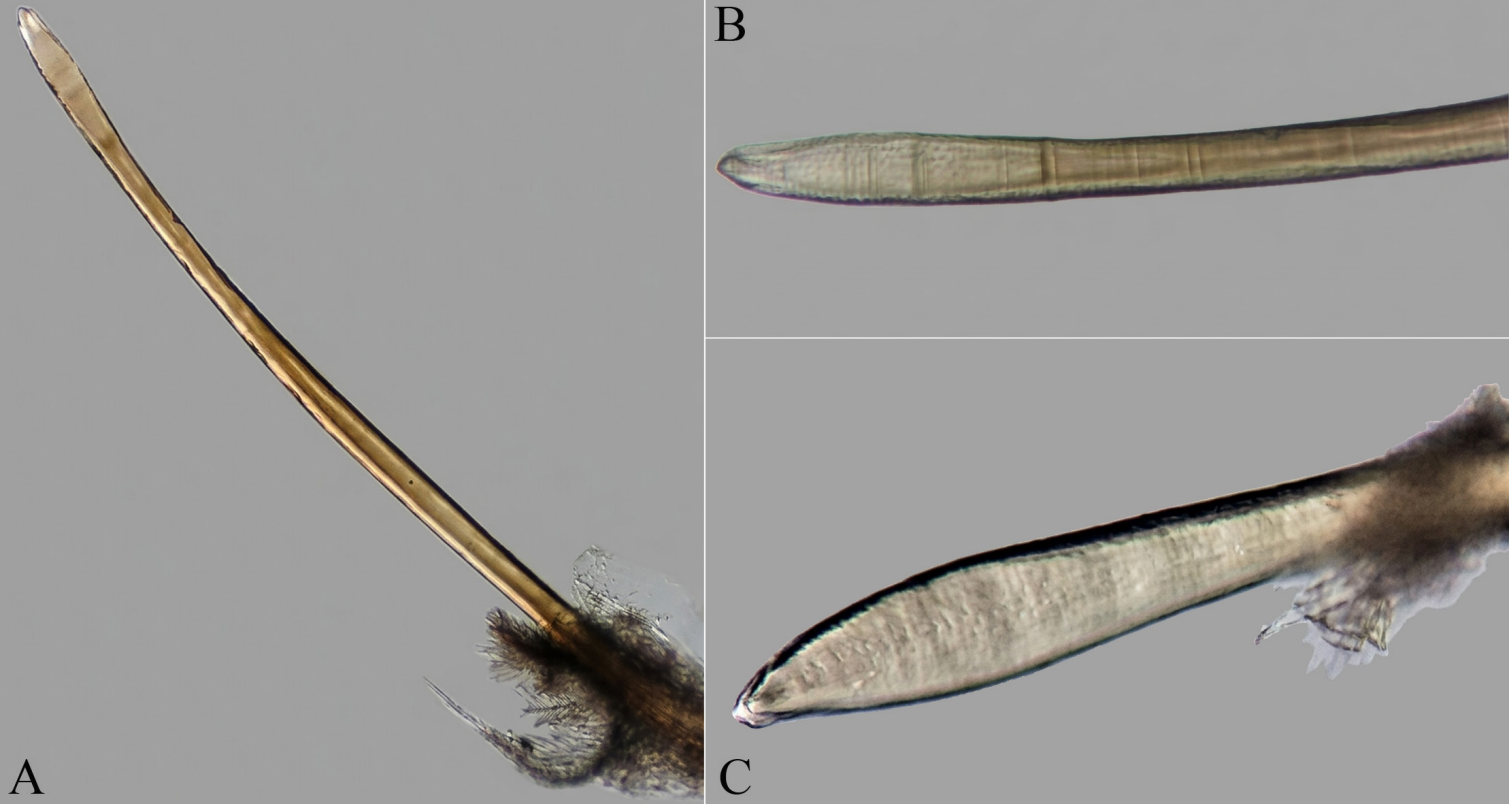
*Kynotus darwinii* (Keller, 1887) genital setae. **A**. Complete seta; **B**. Spoon-shaped tip; **C**. Convex side of tip.

##### Diagnosis.

Aclitellate specimens, 360–375 mm long and 6–7 mm in diameter at segment 16, 3 mm at middle body, and 1.5–2 mm at tail; segment no. 715–730. Colour grey, with slight brownish pigmentation on dorsum at head. Segments 2–13 biannulate. Setae *ab* appear from segment 12, *cd* from 13, male pores large ventral slits on 16. Three rows of small, oval spermathecae in 13/14, 14/15, 15/16, numbered 13–14 pairs in a row (Fig. 1). A large muscular gizzard in 5, septa 5/6, 10/11 slightly thickened 6/7–9/10 strongly strengthened. A pair of large, oval copulatory chambers occupy ventral space of segments 16–18. Each copulatory chamber bears an irregular prostate-like gland (pseudoprostate) that projects dorsally and extends to segment 20. Genital setal glands three pairs in 13, 14, 15. The genital setae are more or less straight with conical apex, length ca 3 mm, diameter 0.05 mm, widened conical apex appears to bear a small depression and with its surface covered with dense, tiny bumps (Fig. 2).

##### Remarks.

*Kynotus darwinii* was described from northwestern Madagascar and later was also reported from Nosy Bé (Michaelsen 1897). Unfortunately, none of the specimens collected so far are adults, so the position of the clitellum for this species is still unknown. Our specimens from northwestern Madagascar match Michaelsen’s description of *K. madagascariensis*, but they are much smaller than Keller’s (1887) specimens of *Geophagus darwinii* (“It reaches a length of 70–80 cm and is 2 cm thick. The colouration is pale bluish red, while the dorsal side of the anterior part of the body is always dark brownish red” Keller 1887: 248). It is possible that Keller (1887) used several species in his description and reported only the size of the largest specimens, because Michaelsen (1897) examined the type specimens and found them identical to his own species, *K. madagascariensis*, which was 230 mm long, but the tail missing.

#### Kynotus insularis

Taxon classification

Animalia

CrassiclitellataKynotidae

Csuzdi & Hong sp. nov.

A06D85F4-6DC2-57BB-893D-59EE45716BF3

https://zoobank.org/694770A7-7D29-497D-98E0-BA6CA533B63D

Figs 3, 4, 5, 6

##### Type material.

***Holotype***: EKCU-Olig-39 (clitellate specimen); Madagascar – Diana District • Ambanja, Nosy Be Island; 13°20'22.96"S, 48°18'10.88"E; 47 m a.s.l.; leg. Y. Hong, M. Razafindrakoto & Solondraza; 13 February 2025. ***Paratypes***: Madagascar – Diana District • EKCU-Olig-40, 1 clitellate adult and 1 praeadult ex.; EKCU-Olig-41, 2 praeadult ex. NIBRIV0000934124 1 praeadult ex., JBNU0009 1 praeadult ex. (GenBank accession no. PZ270348); data the same as holotype • Ambanja, Nosy Be Island; 13°24'03.73"S, 48°17'17.85"E; 17 m a.s.l.; leg. Yong Hong & Malalatiana Razafindrakoto, 14 February 2025.

**Figure 3.**
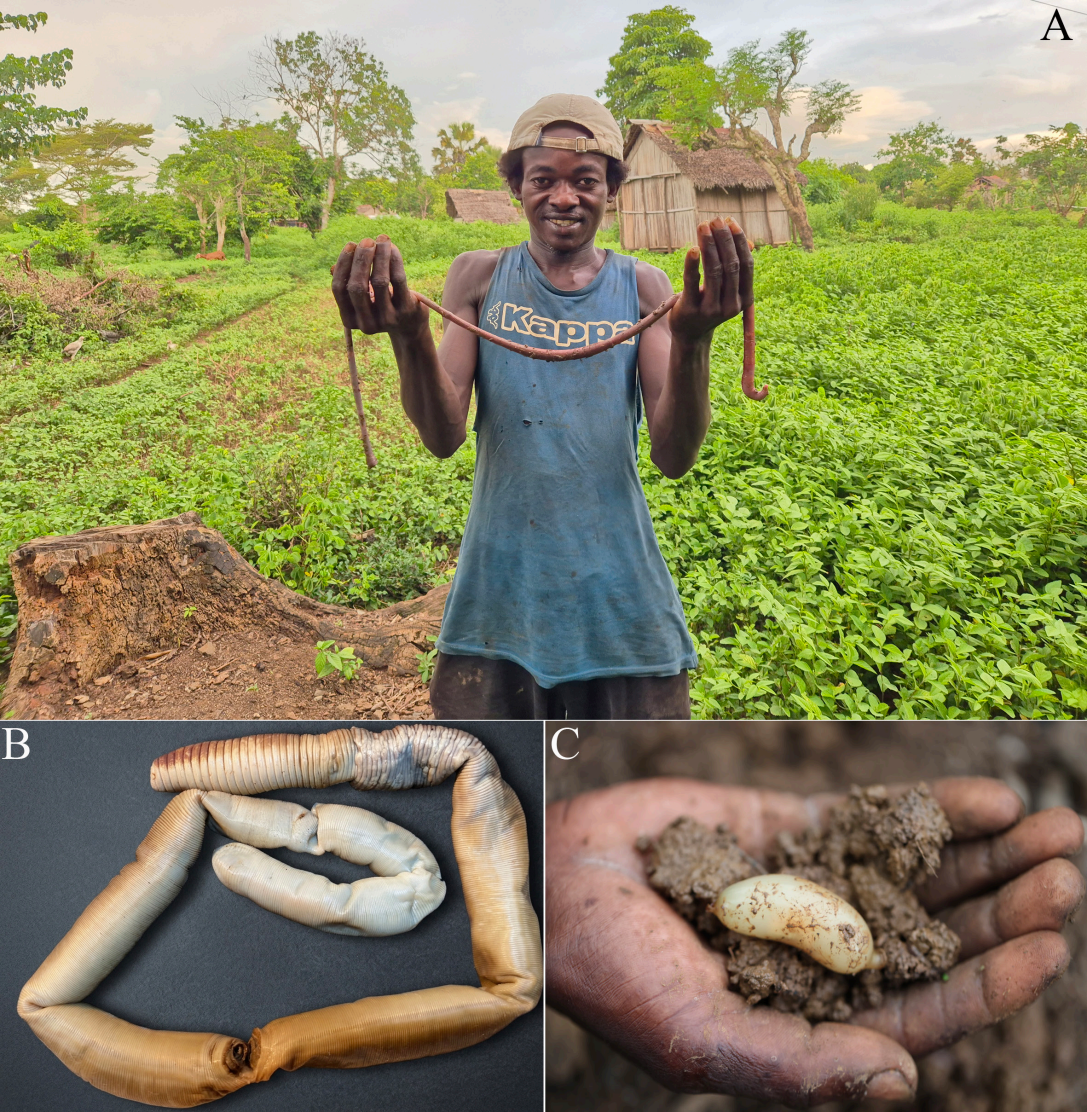
*Kynotus insularis* sp. nov. **A**. Living specimen; **B**. Preserved specimen; **C**. Cocoon.

**Figure 4.**
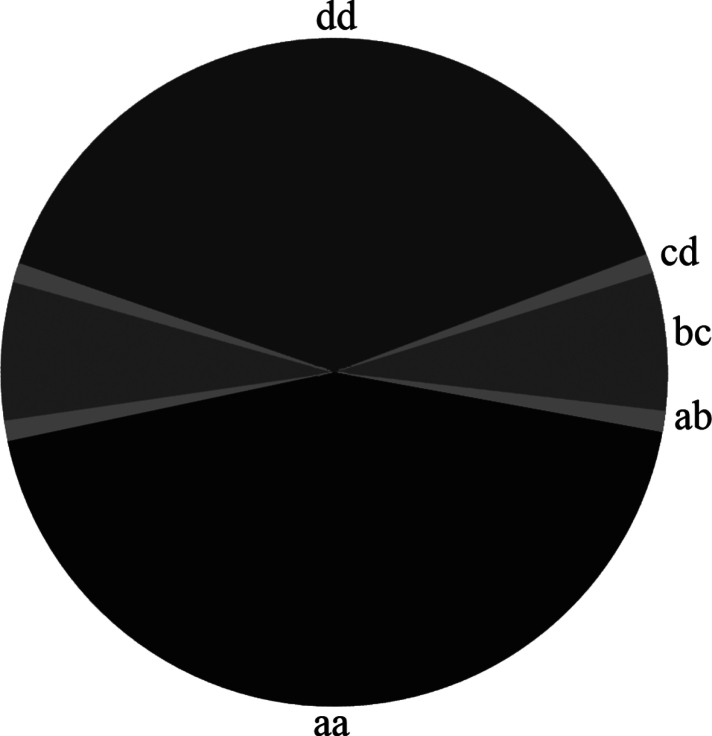
*Kynotus insularis* sp. nov. setal distances.

**Figure 5.**
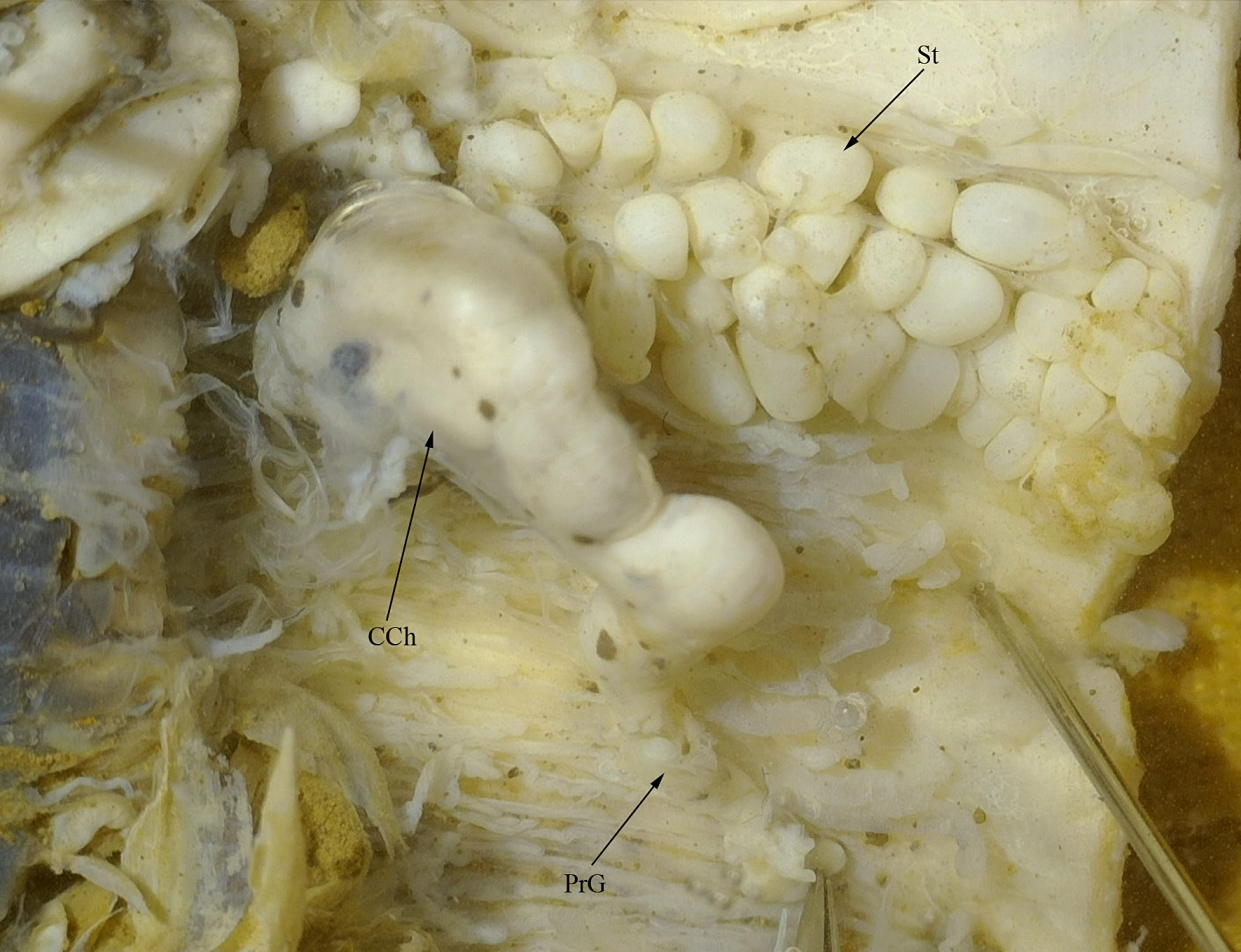
*Kynotus insularis* sp. nov. Spermathecal region. Abbreviations: St = spermathecae, Cch = copulatory cvhamber, Pr.G = prostate-like gland.

**Figure 6.**
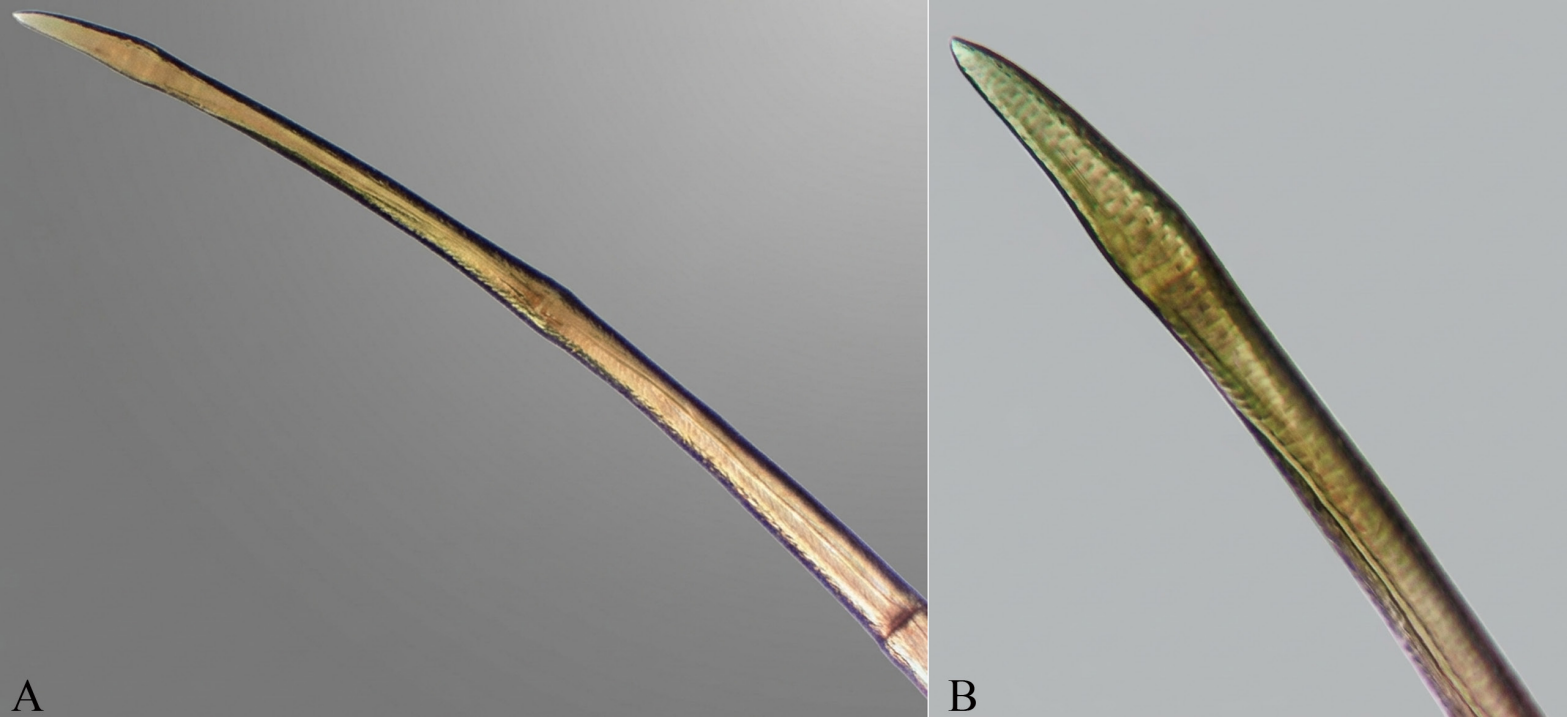
*Kynotus insularis* sp. nov. Genital seta. **A**. Ectal part of the seta; **B**. Lanceolate tip.

##### Etymology.

The specific epithet insularis (adj., 3^rd^ declension) refers to the island habitat (Nosy Be Island) where the species was found.

##### Diagnosis.

Length alive approximately 800 mm, preserved 400–450 mm, diameter 15.5–17.5 mm. Colour dark red on dorsum down to *cd* setal line, pale on ventrum. Segments 1–3 simple, 4–14 biannulate. Setae *ab* and *cd* appear from segments 17 and 15, respectively. Male pores large, ventral on 16. Clitellum circular on segments 21–51. Setae closely paired; *ab* ventrolateral, *cd* lateral. Spermathecal pores from *ab* to M, 10–13 on each side in 13/14, 14/15, 15/16. Septa 6/7–10/11 strongly thickened. Spermathecae elongated, sac-shaped, with a larger ampoule and a small duct. Genital setal glands in three pairs in 13, 14, 15. Genital setae lanceolate, 3.5–3.7 mm in length, with a diameter just under the lanceolate tip of 0.04 mm, just above the nodus of 0.06 mm; ornamentation dense, with small transverse serrations.

##### Description.

***Holotype*** 450 mm in length (preserved); diameter behind clitellum 17 mm; number of segments 873 (tail regenerated). ***Paratypes*** 450–550 mm in length, 13–15 mm in diameter; number of segments 1010–1035. Colour dark red on dorsum, pale on ventrum (Fig. 3). Head prolobous; segments 1–3 simple, 4–13 clearly biannulate. Dorsal pores lacking. Setae small; *ab* and *bc* became observable from segment 17 and 15, respectively. Setae *ab* ventrolateral, *cd* dorsolateral; setal ratio aa:ab.bc:cd:dd = 47.8:1.1:7.3:1:42.5 (Fig. 4). Nephridial pores begin on segment 2, between setal line *ab>cd*. Clitellum complete ring on segments 21–51. Male pores ventral, large oval slits on 16. Female pores not seen. Spermathecal pores in intersegmental furrows 13/14, 14/15, 15/16, 10–13 on each side, between *ab* and M. Genital setal pores in three pairs, segmental on 13, 14, 15.

##### Internal characters.

Large muscular gizzard in 5. Septa 5/6, 11/12 thickened while 6/7–10/11 strongly strengthened. Calciferous glands, lamellae, and typhlosolis lacking. Dorsal blood vessel simple throughout; last pair of hearts in 11. Excretory system holoic, vesiculate, nephridial bladders biramous, with almost equal ental and ectal limbs. Two pairs of testes and sperm funnels in 10, 11 enclosed in free sperm masses. Vesiculae seminales lacking. Ovarium in 13, small longitudinal zigzag fold on the ventral part of the septum 12/13. A pair of large, oval copulatory chambers occupies ventral place of segments 16–22. Each copulatory chamber bears an irregular, prostate-like gland (pseudoprostate) that bulges up to the 30^th^ segment. Spermathecae somewhat elongated, sac-shaped, with small barely discernible duct, 10–13 in each side in segment 14–16 situated from *ab* to the middorsal line (fig. 5). Three pairs of genital setal organs present in segments 13–15, consisting of a small gland similar in shape to pseudoprostates and a genital setal sac containing two mature setae. Genital setae lanceolate, 3.5–3.7 mm in length, with a diameter just under lanceolate tip of 0.04 mm, just above the nodus of 0.06 mm, and ornamented with dense, small, transverse serrations (Fig. 6).

##### Remarks.

The new species is most similar to *K. proboscideus* Razafindrakoto, Csuzdi & Blanchart, 2011 in terms of size, pigmentation, and the segmental position of the spermathecae, but it differs in having a longer clitellum (segments 21–51 vs 22–48), as well as in the shape, number, and position of the spermathecae. Whereas *K. proboscideus* possesses 6–9 elongated, sac-shaped spermathecae, with long stalks opening laterally, the new species is characterised by 10–13 sessile, nearly spherical spermathecae which cover the entire dorsal surface starting from *ab* on both sides. The new species is also somewhat similar to *K. darwinii*, but it differs from it in its much darker pigmentation, size, and the location of the first visible setae, *ab* and *cd*. *Kynotus insularis* sp. nov. clearly differs from both species by its COI barcode as well.

### Barcoding results

**Figure 7.**
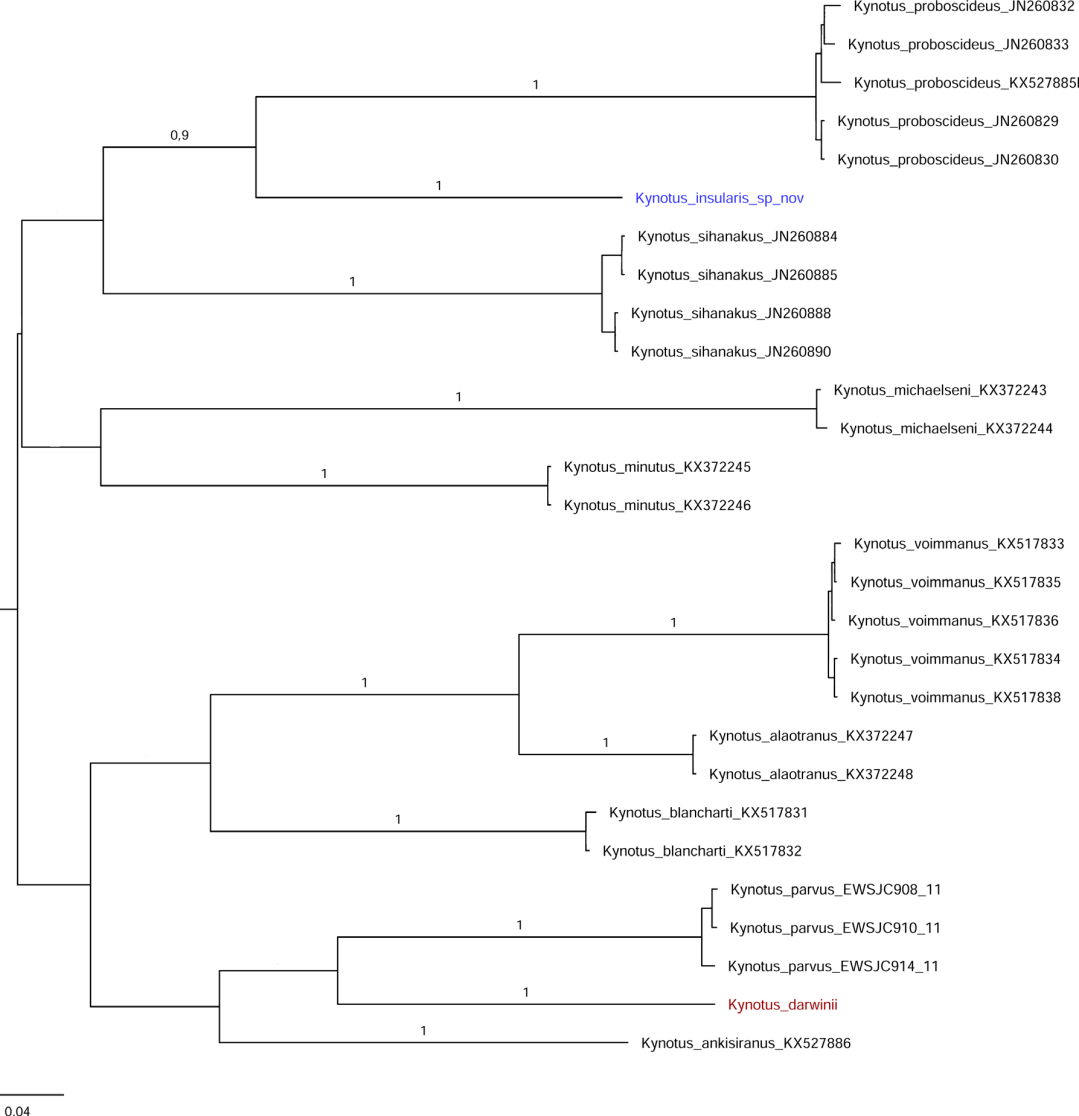
Bayesian tree of the *Kynotus* species based on COI sequences.

**Figure 8.**
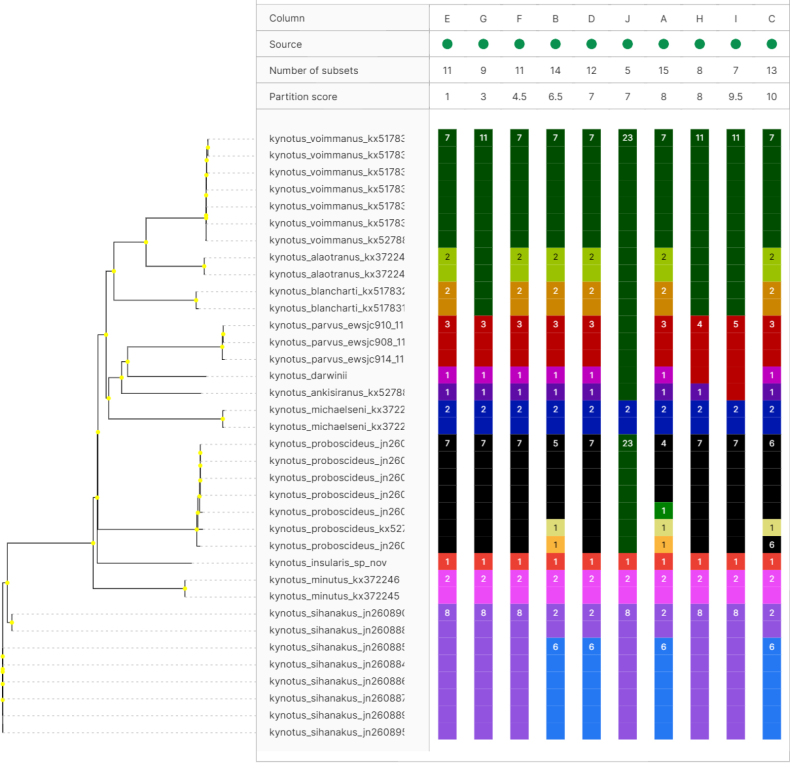
Most supported species partitioning schemes recovered by ASAP (Assemble Species by Automatic Partitioning) based on COI data.

DNA barcoding results are fully congruent with the morphological evidence, and the new species, *K. insularis* sp. nov., forms a well-supported clade with *K. proboscideus* (Fig. 7). However, the genetic distance between these two species is notably high (23.4%). Both their similarity and distinctiveness are further supported by the ASAP analysis, which recovered all morphologically delineated species as the best-supported partition scheme (Fig. 8).

Based on genetic distances (Table 1), *K. darwinii* appears to be most closely related to *K. ankisiranus* (20.3%), and in the Bayesian tree, it forms a weakly supported clade with this species, also including *K. parvus*.

**Table 1.** Pairwise K2P genetic distances of the studied *Kynotus* species.

*K. alaotranus*										
*K. ankisiranus*	0.206									
*K. blancharti*	0.197	0.217								
*K. darwinii*	0.241	0.203	0.215							
*K. michaelseni*	0.255	0.264	0.256	0.253						
*K. minutus*	0.221	0.237	0.224	0.249	0.252					
*K. parvus*	0.268	0.206	0.262	0.207	0.256	0.265				
*K. proboscideus*	0.262	0.260	0.235	0.257	0.282	0.242	0.220			
*K. sihanakus*	0.243	0.233	0.233	0.242	0.261	0.215	0.270	0.233		
*K. insularis*	0.237	0.247	0.239	0.222	0.280	0.234	0.247	0.234	0.223	
*K. voimmanus*	0.143	0.212	0.219	0.261	0.265	0.262	0.272	0.257	0.237	0.244

Overall, K2P genetic distances among the known and sequenced *Kynotus* species are relatively high, generally around or exceeding 20%, with one exception. The only lower value was observed between *K. alaotranus* and *K. voimmanus*, which shows a genetic distance of 14.3%.

### Key to the *Kynotus* species

**Table:** 

1	Spermathecae intramuscular	**2**
–	Spermathecae free, intracoelomic	**3**
2	Spermathecae in 14/15, 15/16; 3–9 on each side/line	***K. friderici* Michaelsen, 1931**
–	Spermathecae 13/14, 14/15, 15/16; 9–14 on each side/line	***K. giganteus* Razafindrakoto et al., 2011**
3	Spermathecae in four lines; 13/14, 14/16, 15/16, 16/17	***K. rosae* Cognetti, 1906**
–	Spermathecae in less than four lines	**4**
4	Spermathecae in two line; 14/15, 15/16	**5**
–	Spermathecae in three line; 13/14, 14/15, 15/16	**9**
5	Spermathecae single in each side	***K. michaelseni* Rosa, 1892**
–	Spermatecae multiple	**6**
6	Two to four spermathecae on each side/line	**7**
–	Spermathecae more numerous	**8**
7	Clitellum on 18–28	***K. pittarellii* Cognetti, 1906**
–	Clitellum on 20–40	***K. sakafotsy* Razafindrakoto et al., 2017**
8	Spermathecae 7–9 on each side/line, setae begin on 17	***K. longus* Michaelasen, 1891**
–	Spermathecae 15–20 or more on each side/line, setae begin on 11	***K. voeltzkowi* Michaelsen, 1897**
9	Single pair of genital gland presents	**10**
–	Two or three pairs of genital glands present	**11**
10	Clitellum on 18–16, ½ 27, colour dark red	***K. minutus* Csuzdi, Razafindrakoto & Blanchart, 2012**
–	Clitellum on 18–28, almost unpigmented	***K. voimmanus* Csuzdi, Razafindrakoto & Hong, 2017**
11	Two pairs of genital glands present in 14, 15	**12**
–	Three pairs of genital glands present in 14, 15, 16	**18**
12	Segments with two ringlet in 4–13	**13**
–	Segment with two ringlets in 4–10	**14**
13	Spermathecae two on each side/line, setae begin on 3	***K. distichotheca* Michaelsen, 1895**
–	Spermathecae 4–6 on each side/line setae begin on 9	***K. sihanakus* Razafindrakoto et al., 2017**
14	Setae begin on 9	***K. sikorai* Michaelsen, 1901**
–	Setae begins on 2 or 3	**15**
15	Spermathecae with well detached long and thin stalk	**16**
–	Spermathecae with short stalk	**17**
16	Red pigmentation on dorsum, genital setae lanceolate	***K. alaotranus* Michaelsen, 1907**
–	Pigmentation lacking, tip of genital setae elongated, spoon-shaped	***K. blancharti* Csuzdi, Razafindrakoto & Hong, 2017**
17	After gizzard several septa thickened, genital setae ca 1 mm long	***K. parvus* Csuzdi, Razafindrakoto & Blanchart, 2012**
–	All septa membranous, genital setae 1.6–1.7 mm long	***K. ankisiranus* Csuzdi, Razafindrakoto & Hong, 2017**
18	Prostomium subdivided, with two small protuberances	**19**
–	Prostomium simple	**20**
19	Clitellum on 22–40, spermathecae 1–3 on each side/line	***K. schistocephalus* Michaelsen, 1897**
–	Clitellum on 22–47, 48 spermathecae 6–9 on each side/line	***K. proboscideus* Razafindrakoto et al., 2011**
20	Setae begins on segment 5	***K. oswaldi* Michaelsen, 1895**
–	Setae begins on segment on 16 or 17	**18**
21	Spermathecae 1–3 on each side/line	***K. kelleri* Michaelsen, 1892**
–	Spermathecae more numerous	**19**
22	Slight red pigmentation on dorsum, setae *ab* begin on 12	***K. darwinii* Keller, 1887**
–	Dark red pigmentation to *cd*, setae *ab* begin on 17	***K. insularis* sp. nov**.

## Discussion

As a result of our ongoing research, our understanding of the Malagasy earthworm fauna continues to expand. However, we are far from finished with our research. This is clearly illustrated by our recent expedition to northern Madagascar, which resulted in the discovery of a new *Kynotus* species and the rediscovery of a little-known one. With these additions, the number of Malagasy endemic Kynotidae species has increased from 22 (Csuzdi 2012) to 23.

Barcode sequences are currently available for 11 of these species and, apart from a single exception, they exhibit strong species-specificity. Only the genetic distance between the *K. voimmanus*–*K. alaotranus* species pair approaches the threshold proposed by Chang and James (2011) (15% K2P divergence). However, these species can be readily distinguished morphologically by the larger body size and strongly thickened anterior septa of *K. voimmanus*.

It is also noteworthy that nine of the 23 *Kynotus* species have not yet been recollected during our recent field efforts, further underscoring the incomplete state of our knowledge and the need for continued exploration of the Malagasy earthworm fauna.

## Supplementary Material

XML Treatment for Kynotus darwinii

XML Treatment for Kynotus insularis

## Additional information

### Artificial Intelligence (AI) use

The authors accept full responsibility for the content of the manuscript, including the disclosure of any use of AI.

Regarding the use of AI in the preparation of this manuscript, the authors declare the following: Gemini

Used for: Figures, tables and charts

The background of the micrograps were cleared by using Gemini 3 AI as stated in the M & M.

### Author ORCIDs

C. Csuzdi https://orcid.org/0000-0002-0319-7836

M. Razafindrakoto https://orcid.org/0000-0002-4596-4319

Y. Hong https://orcid.org/0000-0002-8093-9717

### Data availability

All of the data that support the findings of this study are available in the main text.
